# Innovative Aqueous Nanoemulsion Prepared by Phase Inversion Emulsification with Exceptional Homogeneity

**DOI:** 10.3390/pharmaceutics15071878

**Published:** 2023-07-04

**Authors:** Patrícia C. Pires, Mariana Fernandes, Francisca Nina, Francisco Gama, Maria F. Gomes, Lina E. Rodrigues, Sara Meirinho, Samuel Silvestre, Gilberto Alves, Adriana O. Santos

**Affiliations:** 1 CICS-UBI—Health Sciences Research Centre, University of Beira Interior, Av. Infante D. Henrique, 6200-506 Covilhã, Portugal; patriciapires93@gmail.com (P.C.P.); mariana-96@live.com.pt (M.F.); francisca.nina7@gmail.com (F.N.); franciscogama98@gmail.com (F.G.); mfsfgomes@hotmail.com (M.F.G.); linaisabelestevesrodrigues@gmail.com (L.E.R.); sara.alexandra92@gmail.com (S.M.); samuel@fcsaude.ubi.pt (S.S.); gilberto@fcsaude.ubi.pt (G.A.); 2 Department of Pharmaceutical Technology, Faculty of Pharmacy, University of Coimbra, 3000-548 Coimbra, Portugal; 3 Associated Laboratory for Green Chemistry (LAQV) of the Network of Chemistry and Technology (REQUIMTE), Faculty of Pharmacy, University of Coimbra, 3000-548 Coimbra, Portugal; 4 Faculty of Health Sciences, University of Beira Interior, Av. Infante D. Henrique, 6200-506 Covilhã, Portugal; 5 Faculty of Sciences, University of Beira Interior, Rua Marquês d’Ávila e Bolama, 6201-001 Covilhã, Portugal

**Keywords:** nanoemulsion, intranasal, low energy, homogenous, phase inversion, propylene glycol monocaprylate type II

## Abstract

Formulating low-solubility or low-permeability drugs is a challenge, particularly with the low administration volumes required in intranasal drug delivery. Nanoemulsions (NE) can solve both issues, but their production and physical stability can be challenging, particularly when a high proportion of lipids is necessary. Hence, the aim of the present work was to develop a NE with good solubilization capacity for lipophilic drugs like simvastatin and able to promote the absorption of drugs with low permeability like fosphenytoin. Compositions with high proportion of two lipids were screened and characterized. Surprisingly, one of the compositions did not require high energy methods for high droplet size homogeneity. To better understand formulation factors important for this feature, several related compositions were evaluated, and their relative cytotoxicity was screened. Optimized compositions contained a high proportion of propylene glycol monocaprylate NF, formed very homogenous NE using a low-energy phase inversion method, solubilized simvastatin at high drug strength, and promoted a faster intranasal absorption of the hydrophilic prodrug fosphenytoin. Hence, a new highly homogeneous NE obtained by a simple low-energy method was successfully developed, which is a potential alternative for industrial application for the solubilization and protection of lipophilic actives, as well as (co-)administration of hydrophilic molecules.

## 1. Introduction

Most drug candidates are highly lipophilic in nature, making their high strength solubilization in liquid dosage forms a challenge. Additionally, most drugs are easily susceptible to chemical and/or enzymatic degradation, which reduces their bioavailability at the action site. To tackle these issues, several strategies have been developed, with the encapsulation of molecules in nanosystems being one of the most successful [1,2].

Nanosystems are typically classified as colloidal structures with a size of less than 500 nm, inside which drugs are incorporated. This will allow not only to increase the preparation’s drug strength, but also to protect the molecules from degradation, increase their permeability through biological membranes, promote bioavailability, reduce plasma protein binding, and allow a controlled drug release, with the possibility of targeting certain organs. There are many types of nanosystems, polymeric and lipid nanosystems being the most frequently used. There is a great variety of lipid nanosystems: liposomes and derived structures (transfersomes, niosomes, cubosomes, ethosomes, etc.), solid lipid nanoparticles, nanostructured lipid carriers, and nanometric emulsions [3,4]. Nevertheless, many of these nanocarriers have important disadvantages, such as being complex, time-consuming and having polluting preparation methods, low physical stability, low encapsulation efficiency, and lack of biocompatibility. However, these disadvantages are reduced or simply do not apply to nanometric emulsions, particularly those with the right formulas, which have good stability, solubilization capacity, and simple preparation methods.

Nanometric emulsions include nanoemulsions (NE) and microemulsions. Microemulsions, named as such since 1959 [5], are thermodynamically stable and spontaneously form liquid dispersions of lipids and water, generally implying high amounts of surfactants and co-solvents/co-surfactants [6,7]. The droplet size is usually inferior to 100 nm, often not much larger than simple surfactant micelles, in which case they may appear completely transparent. The nature of their composition makes them related to classic emulsions, which are, however, biphasic thermodynamically unstable systems.

In turn, NE are colloidal liquid-in-liquid dispersions, usually presenting a size below 200 nm and a higher kinetic stability than macroemulsions. Compared with microemulsions, NE are usually composed of a lower content of hydrophilic surfactants and co-solvents, and usually require high energy methods for shearing and homogenizing the lipid droplets. Nevertheless, specific NE compositions can also be prepared by low energy methods, but with some compromise in the amounts of surfactant and co-solvents that are used (which tends to be high, although not as much as in microemulsions), and in the degree of homogeneity, which is tendentially low [8].

Oil-in-water NE are a good strategy to deliver solubilized lipophilic drugs at high drug strengths. This is of high importance, especially for some delivery routes, such as in intranasal administration to the systemic circulation or to the brain, given the relatively short residence time and small volume of administration [9,10,11,12].

Hence, the purpose of this work was to develop a NE using low amounts of surfactants and/or cosolvents, thus with high lipid content and good solubilization of lipophilic drugs like simvastatin, while able to promote the absorption of drugs with low permeability like fosphenytoin. At the same time, it was aimed at a homogenous size under 200 nm and, preferably, a low energy preparation method. We screened NE compositions with a high proportion of relatively polar lipids aiming to achieve a good solubilization of drugs such as simvastatin and then characterized their droplet size. A composition with low proportion of hydrophilic surfactant to oils and no co-solvent originated a NE about 100 nm in droplet size using a simple low energy phase inversion method. Surprisingly, these NE spontaneously became highly homogenous upon refrigeration, with a polydispersity index (PDI) < 0.1, which is not usually found in NE without homogenization. Therefore, we secondarily aimed to establish which formulation factors were required for this attribute. The extremely low PDI was lost when changing excipients to similar ones or by changes in their proportion outside a narrow range. We also describe alternative compositions of the aqueous phase in which the NE display PDI < 0.1 already at room temperature. Given the high lipid content of such NEs, a high drug strength of lipophilic substances was obtained. Furthermore, the intranasal administration of the NE of a hydrophilic prodrug (fosphenytoin) to mice demonstrated a faster absorption than an aqueous solution of the drug.

## 2. Materials and Methods

### 2.1. Materials and Reagents

The hydrophilic surfactants Kolliphor^®^ RH 40 (Macrogolglycerol hydroxy stearate), Kolliphor^®^ EL (Macrogolglycerol ricinoleate), Kolliphor^®^ P124 (Poloxamer 124), Kolliphor^®^ HS 15 (Polyethylene glycol 660 12-hydoxystearate), and the oil Kollicream^®^ IPM (Isopropyl Myristate) were kindly offered by BASF (Ludwigshafen, Germany); Transcutol^®^ HP (Diethylene glycol monoethyl ether), a cosolvent, Labrasol^®^ ALF (Caprylocaproyl polyoxyl-8 glycerides), a hydrophilic surfactant, and the oils Capryol^®^ 90 (Propylene Glycol Monocaprylate (type II) NF), Capryol^®^ PGMC (Propylene glycol monocaprilate, Type I), LabrafacTM PG (Propylene glycol dicaprylocaprate), Maisine^®^ CC (Glycerol monolinoleate), and PeceolTM (Glycerol mono-oleate) were kindly offered by Gattefossé (Saint-Priest, France); the oils Imwitor^®^ 948 (Glyceryl mono-oleate), Imwitor^®^ 988 (Glycerol monocaprilate, Type I), and Softisan^®^ 64S (Bis-diglyceryl polyacyladipate-2) were kindly offered by IOI Oleo GmbH (Hamburg, Germany); Capmul^®^ MCM (Glycerol monocaprilocaprate), Capmul^®^ 808G EP/NF (Glycerol monocaprylate Type II), Capmul^®^ PG-8 (Propylene glycol monocaprylate), and Capmul^®^ PG-8-70 NF (Propylene glycol monocaprylate Type II) were kindly offered by Abitec; Miglyol^®^ 812 (medium-chain triglycerides; Caprylic/Capric Triglyceride), Soybean oil, Span^®^ 80 (Sorbitane mono-oleate), Vitamin E Acetate, Cetiol V (Decyl oleate), the hydrophilic surfactants Tween^®^ 20 (Polysorbate 20) and Tween^®^ 80 (polysorbate 80), and the polymers Polyethylene glycol (PEG) 4000, (Hydroxypropyl)methyl cellulose (HPMC, corresponding to Hypromellose Viscosity 4000 mPa·s), and Polyvinylpyrrolidone (PVP, corresponding to Povidone K30) were acquired from Acofarma^®^ (Madrid, Spain); the surfactant Tyloxapol was from Acros Organics (Thermo Fisher ScientificGeel, Belgium); Malic acid was acquired from Applichem (Darmstadt, Germany). Ultra-pure water was obtained from a Mili-Q^®^ purification system from Millipore (Billerica, MA, USA). Simvastatin (98.03% purity) was purchased from Bld Pharmatech GmbH. (Kaiserslautern, Germany.) and kept at 4 °C under a nitrogen atmosphere during utilization. The bovine serum albumin (BSA) was acquired from Sigma-Aldrich, Inc (St. Louis, MO, USA). Acetonitrile and methanol were high-performance liquid chromatography (HPLC) gradient grade. The symbols TM and^®^ will be omitted from now on for simplification.

### 2.2. Nanoemulsions Preparation

The oil phase (preconcentrate) was prepared by weighing the lipids and surfactants and mixing them from a few seconds to a few minutes until a homogenous solution was obtained. Depending on the NE, the aqueous phase either consisted in water, a 30 mM pH 5 malate buffer, or a 20 mM pH 7 phosphate buffer, to which NaCl, BSA, PEG 4000, HPMC, or PVP were added at the indicated concentrations (presented with the respective data in the results section). To prepare the NE, a phase inversion method was used, in which about a quarter of the final aqueous phase mass was first added and mixed with e preconcentrate, followed by the addition and mixture of the remaining aqueous phase mass.

### 2.3. Nanoemulsion’s Droplet Size and Zeta Potential

Both hydrodynamic diameter (droplet size) and PDI were measured by dynamic light scattering (DLS) technique associated with cumulants analysis. For that, a Zetasizer Nano ZS (Malvern^®^, United Kingdom) combined with the Zetasizer software (version 7.10) was used. Before each measurement, samples were diluted, about 500-fold, in ultra-pure water. For each tested formulation, two independent discardable cuvettes were prepared and three different measurements of each were automatically performed by the equipment set either at 20 or 25 °C. Measurements were typically performed within 30 min after dilution. As measurement parameters set in Zetasizer software, water was considered as the dispersant (Refractive Index = 1.330 and Viscosity = 0.8872 cP) and the material was set to a Refractive Index = 1.450, representing “lipid”). When assessing the role of refrigeration in mean droplet size and PDI, formulations were placed at 4 °C overnight (at least 12 h), and then measurements were performed exactly as stated before, after diluting the samples immediately upon removing them from the refrigerator.

Zeta potential was measured with Malvern’s Dip Cell Kit in the same equipment, using the same preparation steps. Measurements were taken at 20 or 25 °C and water was selected as the dispersant (Dielectric Constant = 78.5) and “lipid” as the material.

### 2.4. Osmolality

Osmolality was measured using Osmomat^®^ 3000 freezing point osmometer from Gonotec^®^ GmbH (Berlin, Germany). Osmolality measurement was performed in independent triplicates. The device was previously calibrated using ultra-pure water and two standard solutions of 300 mOsmol/Kg and 850 mOsmol/Kg.

### 2.5. Viscosity

Viscosity measurements were performed at different rotational velocities in a Brookfield DV3T^TM^ RV Cone Plate (DVTRVCP) Rheometer (Toronto, ON, Canada), using the CPA-40z cone spindle (viscosity range of 1.7–32,700 cP) and the Rheocalc T^®^ software (version 1.1.13). Measurements were performed at controlled temperature using a thermostatic water bath (MultiTemp III Thermostatic Circulator, Thermo Fisher Scientific, Waltham, MA, USA). Before viscosity measurements, equipment calibration was verified using Ametek Brookfield Fluid 500 Viscosity Standard (Middleborough, MA, USA) with a standardized viscosity of 489 cP at 25 °C. At each velocity, viscosity was registered after the spindle had enough time to perform five complete rotations. For fluids with Newtonian rheological behavior, viscosity was determined at the shear rate corresponding to the highest torque value (just below 100%), due to the equipment’s higher resolution and precision resulting in a lower measurement error. For non-Newtonian fluids, zero shear viscosity was estimated by the Y-intercept of the non-linear regressions based on the measurements performed at different shear rates at a constant temperature.

### 2.6. In Vitro Release of Model Drug

In vitro drug release studies, performed in horizontal Ussing Chambers (Harvard Apparatus, NaviCyte, Hugstetten, Germany), used phenytoin as a model drug. The methodology was adapted from Pires et al. [13]. Synthetic membranes were used, with a pore size of 0.2 μm (hydrophilic polyethersulfone Supor^®^ membrane disc filters, Pall Life Sciences, MI, USA). The receiving chamber was filled with 1.8 mL of phosphate buffer (pH 7, 20 mM), plus albumin at 1% *w*/*w*, and 100 μL of this same solution was placed on the donor side of the membrane. The temperature was kept at 32 °C, the approximate temperature of the nasal cavity at the region of the middle turbinate [14], with a heating water bath (Grant Instruments, Cambridge, England), and receiving chamber homogenization was achieved through magnetic steering (Micro Stirring Bars, 2 mm, VWR, United Kingdom). When the intended temperature was reached, the buffer on the donor chamber was replaced with 100 μL of the test formulations. Afterwards, samples of 100 μL were taken from the receiving chamber and replaced by the same volume of buffer plus albumin solution at 10, 20, 40, 60, 90, 120, 180, and 240 min.

For phenytoin quantification, samples were diluted 5-fold in a water-Transcutol mixture (3:1), followed by addition of perchloric acid at 10% (*v*/*v*). Formulation’s initial drug concentration was also quantified in a similar way, but the dilution was 500-fold. A previously developed and validated HPLC method [15] was used, comprising specific apparatus and conditions: LC-2010A HT Liquid Chromatography system, coupled with a SPD-M20A diode-array detector, controlled by LabSolutions (version 5.52) software (Shimadzu, Kyoto, Japan); analyte separation was performed in a reversed-phase column (3 μm particle size, 55 × 4 mm) protected by a guard column (5 μm particle size, 4 × 4 mm, C18, LiChroCART^®^ Purospher^®^ STAR, Merck, Darmstadt, Germany), with isocratic elution at 1 mL/min at 30 °C. Mobile phase was a mixture of 36% (*v*/*v*) methanol and 64% (*v*/*v*) sodium phosphate buffer (10 mM, pH 3, with 0.25% triethylamine), filtered (Nylaflo membrane, 0.2 μm pore size, Pall, NY, USA) and degassed in a ultrasound bath. Sample injection volume was 20 μL, and phenytoin was detected at 215 nm, with a retention time of 10–11 min, within 20 min runs.

### 2.7. Cell Culture and Cytotoxicity Evaluation

Normal Human Dermal Fibroblasts (NHDF, adult donor cells, Ref. C-12302 from PromoCell) were cultured in RPMI 1640 medium supplemented with inactivated fetal bovine serum at 10%, 2 mM *L*-glutamine, 10 mM 4-(2-hydroxyethyl)-1-piperazineethanesulfonic acid, 1 mM sodium pyruvate, and 1% antibiotic (10,000 U/mL penicillin G, 100 mg/mL streptomycin) at 37 °C in a cell incubator with a humidified atmosphere of 5% carbon dioxide. The culture media was renewed every two or three days, and cells were subcultured as required.

To evaluate formulations’ cytotoxicity, 15,000 cells were seeded in 100 µL per well of 96-well culture plates the day preceding the experiment. Immediately before the experiment, the medium was replaced by 50 µL of complete fresh medium plus 50 µL of NE preconcentrates diluted in fresh medium at twice the concentration selected for treating the cells. Cells were then incubated for 30 min, after which the treatment medium was replaced by 200 µL of 10% (*w*/*v*) solution of resazurin prepared in Krebs Ringer Buffer (NaH_2_PO_4_.H_2_O 1.5 mM; Na_2_HPO_4_ 0.83 mM, KCl 4.86 mM; NaCl 119.78 mM; MgCl_2_.6H_2_O 1.67 mM; NaHCO_3_ 15 mM; anhydrous *D*-Glucose 10 mM; CaCl_2_.2H_2_O 1,2 mM) and plates incubated at 37 °C for the required time (until negative control wells had approximately a reference fluorescence level—10,000 RFU in our plate reader, as previously optimized). The fluorescence intensity of plate wells was measured using an excitation wavelength of 570 nm and emission wavelength of 590 nm in a microplate fluorophotometer (Spectramax GeminiTM EM, Molecular Devices, LLC, San Jose, CA, USA). After subtracting background fluorescence (wells with blank resazurin solution), cell viability data were expressed as a percentage of the negative control of non-treated cells. Each experiment was carried out in quadruplicate wells.

### 2.8. In Vivo Pharmacokinetic Study of Model Drugs through Intranasal or Subcutaneous Administration

Healthy adult CD-1 mice (8 to 11 weeks old, 39 to 42 g), originated from Faculty of Health Sciences’ certified animal facility were housed with controlled environmental conditions (12 h light/dark cycle, 20 ± 2 °C, 50 ± 5% relative humidity). Mice had free access to acidified tap water and standard rodent diet. All experimental procedures were carried out in conformity with the regulations of the European Directive 2010/63/EU and approved both by the Local Animal Ethics Committee and the competent national authority [Portuguese National Authority for Animal Health, Phytosanitation and Food Safety (Direção Geral de Alimentação e Veterinária)].

Mice were randomly divided into 2 groups, receiving either intranasal or subcutaneous administration (12 animals, 3 time points, 2 mice per time point). All animals were anesthetized with an intraperitoneal dose of pentobarbital (60 mg/kg) prior to formulation administration. All administrations were carried out with the mouse’s body lying on top of a heating pad (plus a DC Temperature Controller 40-90-8D, FHC, Bowdoin, ME, USA). The established administration volume was of 5 μL and 50 μL per 30 g of body weight, for intranasal and subcutaneous administrations, respectively. Afterwards the mice were left to recover in a supine position, in a temperature-controlled environment.

Euthanasia was conducted at specific time points (30, 240 and 720 min), after which mice blood and brain were collected. The blood was collected into K3 EDTA tubes (FL Medical, Italy), after which 300 μL were mixed with orthophosphoric acid 85% (*v*/*v*) in a 1:1 (*v*/*v*) blood/acid ratio. The brains were homogenized (Ika Ultra-Turrax^®^ T25 Basic, Staufen, Germany) in a water-orthophosphoric acid [1:1 (*v*/*v*)] mixture (1 g of tissue per 4 mL of mixture), centrifuged (14,000 rpm, 4 °C, for 10 min, MIKRO 200R microcentrifuge, Hettich, Tuttlingen, Germany), and the supernatants were collected. Both acidified blood and brain homogenates were kept on ice and then stored at −20 °C.

Sample processing consisted of the addition of 20 μL of ketoprofen spiking solution (internal standard) to the sample (100 μL of acidified brain homogenate supernatant or 200 μL of acidified blood), followed by liquid–liquid extraction [addition of 1000 μL of diethyl ether, followed by vortexing for 30 s, and then by centrifugation for 5 min at 13,500 rpm at room temperature in a tabletop microcentrifuge (Gyrozen, Daejeon, Republic of Korea)]. Next the organic phase was transferred to a glass tube, and the aqueous phase was reextracted two more times, under the same conditions. The combined organic phases were then evaporated to dryness (gas stream, 45 °C) and reconstituted with 100 μL of mobile phase. The mobile phase was made of 36% (*v*/*v*) methanol and 64% (*v*/*v*) sodium acetate buffer (10 mM, pH 5, with 0.25% triethylamine). In each sample, phenytoin quantification was performed by HPLC, using a previously developed and validated method [15], and aside from a change in mobile phase composition most chromatographic apparatus and analyte separation conditions were the same as described in Section 2.6. for the drug release study. The only other parameter that was modified was the analytes’ detection wavelength, which remained 215 nm for phenytoin and fosphenytoin, but was changed to 280 nm for the internal standard.

### 2.9. Statistical Analysis

Data are represented by means of replicate measurements or of independent formulations ± standard deviation. Cell viability data were fitted by a nonlinear regression model [log(inhibitor) vs. normalized response—Variable slope] using GraphPad Prism version 9.5.1 to determine half-inhibitory concentrations (IC_50_).

## 3. Results

### 3.1. A Spontaneously Formed Nanometric Emulsion with Exceding Homogeneity

The purpose of this study was to develop NE with good solubilization capacity for several drugs with low water solubility, such as simvastatin and phenytoin. For that, we selected two relatively polar lipids (monoesters of propylene glycol or glycerol), Kolliphor RH 40 as surfactant and Transcutol as co-surfactant, further testing several compositions and proportions (Figure S1). NE were prepared by adding water in two steps to the mixture of organic excipients followed by homogenization by premix membrane emulsification. However, one of the tested formulas originated an emulsion already quite homogenous, prior to any homogenization (PDI close to 0.2, Figure S1), with a mean size lower than 200 nm, which was surprising given that it had a high lipid proportion and no co-surfactant. Furthermore, after refrigeration, that formulation became noticeably less opaque, suggesting a reduction in droplet size, a characteristic that reverted upon thawing back to room temperature.

**Figure 1:**
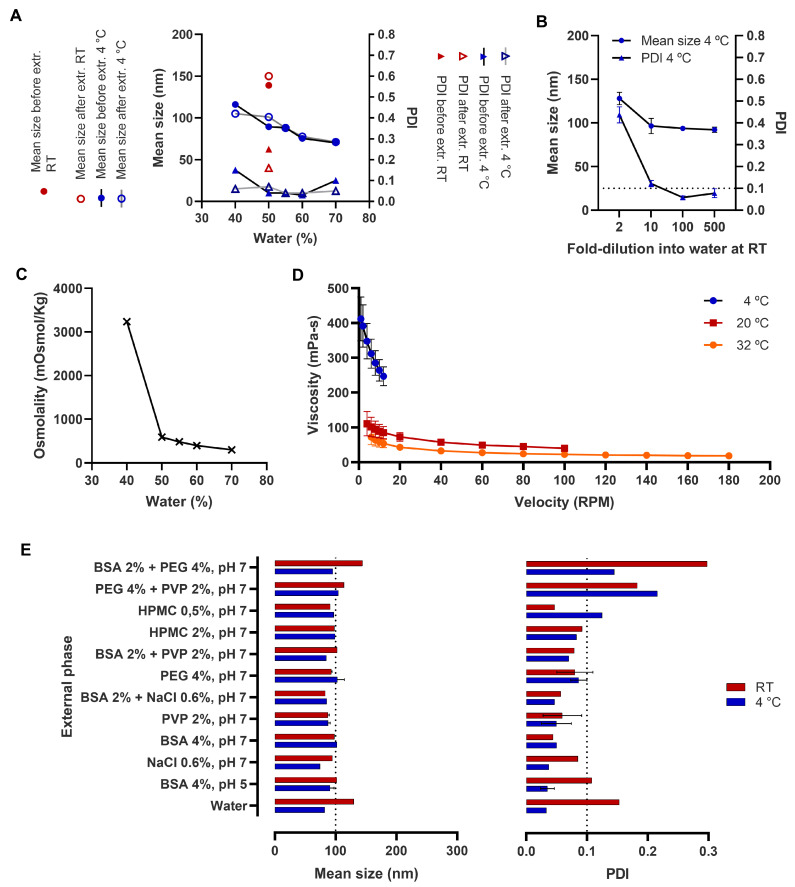
Characterization of the emulsions prepared from the selected preconcentrate with different aqueous phase proportions and composition. (**A**) Water proportion and temperature effect on the mean droplet size and polydispersity index (PDI) of the emulsions; (**B**) Water proportion effect on emulsion’s osmolality; (**C**) Dilution effect of refrigerated concentrated emulsions (50% water) at room temperature (RT) water before measurement of the mean droplet size and PDI of the emulsions; (**D**) Viscosity of the nanoemulsion prepared with 50% aqueous malate buffer pH 5, at different temperatures; (**E**) Effect of the addition of different hydrophilic polymers and salt on the mean droplet size and PDI of the emulsions. Data corresponds to mean of replicate measurements/cuvettes (**A**,**C**,**E**) or mean ± standard deviation of four independent dilutions (**B**) or three independent formulation baches (**D**). Some of the data shown in (**E**) is mean ± standard deviation of two independent formulation baches.

To further understand this phenomenon, the effect of temperature on droplet size was thoroughly studied. It was found that upon 500-fold dilution for characterization by DLS, the temperature of the water and the temperature used during measurements was irrelevant (not shown). In fact, the factor with real impact on NE droplet size was the temperature of the concentrated emulsion at the time of its dilution into room temperature water, and whenever a temperature is mentioned (e.g., Figure 1A,B,E), it is the temperature of the concentrated emulsion before dilution into water at room temperature. At room temperature, membrane pre-emulsification had no relevant effect on the size of emulsions with 50% water content, only promoting a slight reduction in PDI from over 0.2 to slightly under 0.2 (Figure 1A). However, upon refrigeration, the exact same NE suffered a significant reduction in droplet mean size to around 100 nm, and in PDI to under 0.1, with homogenization being irrelevant (Figure 1A). This effect was not solely related to time since emulsions left overnight at room temperature did not become more homogeneous and thawing them back to room temperature after refrigeration reverted the droplet size (not shown). The authors are not aware of a similar phenomenon being previously described in the literature.

In refrigerated NE, an increase in water content from 40% to 70% seemed to reduce droplet size. In NE containing between 50% and 60% water content, PDI was under 0.1 without membrane homogenization (Figure 1A). At least a 10-fold dilution appears to be required for refrigerated emulsions to maintain their low size and PDI (Figure 1B).

Formulations were slightly hypertonic but within a safe osmolality interval in the water range of 50–70% (Figure 1C), moderately viscous (up to around 100 mPa·s) at room temperate, and significantly more viscous after refrigeration (up to about 400 mPa·s, Figure 1D).

Next, we evaluated the effect of adding different hydrophilic polymers to the aqueous phase and found that several of them induced NE to have a droplet size under 100 nm and PDI under 0.1 without refrigeration (Figure 1E). This was the case with PVP, BSA (at pH 7 but less so at pH 5), HPMC at 0.5%, and PEG 4000.

### 3.2. The Very High Homogeneity in Nanoemulsion’s Is Easily Lost by Small Changes in Preconcentrate Composition

**Figure 2:**
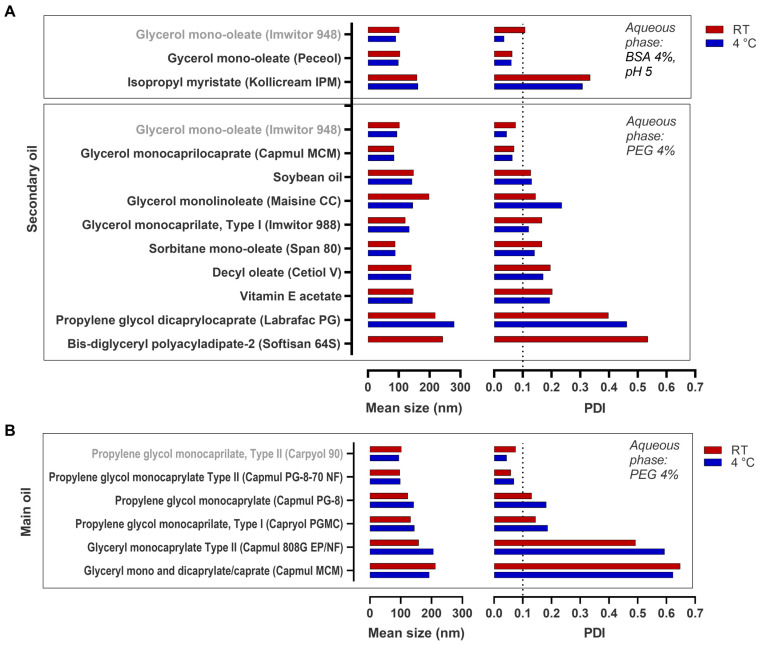
Mean droplet size characterization of emulsions obtained by changing one of the hydrophobic excipients in the selected preconcentrate. (**A**) Secondary oil and temperature effect on the mean droplet size and polydispersity index (PDI) of emulsions; (**B**) Main oil and temperature effect on the mean droplet size and PDI of emulsions. Data correspond to the mean of at least two independent dilutions of the same nanoemulsion.

To better understand which excipients of the present formula were related to the favorable size characteristics of the NE, several other formulas were screened by replacing each of the excipients with others of the same class. Concerning the minoritarian oil, glycerol mono-oleate (Imwitor 948), it was found that it could be replaced by another brand of glycerol mono-oleate (Peceol) or by glycerol monocaprylocaprate (Capmul MCM), but not by the closely related monolinoleate (Maisene CC) or any of the other tested lipids (Figure 2A).

It was found that Capryol, 90, the oil present in NE at higher proportion, could only be replaced by another brand of the exact same variety of propylene glycol monocaprylate type II—Capmul PG-8-70 NF—but not by other propylene glycol monocaprylates (type I or a variety not following pharmacopeia’s specification), neither by glyceryl monocaprylates (Figure 2B).

The relative cytotoxicity of NE with different compositions was screened by exposing human fibroblasts (NHDF) cells to different preconcentrate concentrations (0.0016–0.16% or 0.16–45 or 50%) for 30 min. The obtained IC_50_ of the preconcentrate of the reference NE (without any drug) was about 0.04%. In fact, both Capryol 90 and Imwitor 948 are approved for cutaneous and oral administration, but not for parental use. Changing Imwitor 948 for other lipids did not significantly reduce formulation’s cytotoxicity (all IC_50_ were ≤0.06%) (Table 1).

To better rank the tested lipids according to their cytotoxicity, they were solubilized by mixing with Kolliphor RH 40:Transcutol:water (1:4:4:1), forming a self-microemulsifying preconcentrate, followed by cytotoxicity evaluation using the same method (Table 2). This allowed to rank several lipids from the less toxic to the more toxic: Vitamin E Acetate; Miglyol 812; Softisan 64S < Labrafac PG; Kollicream IPM; Maisine CC < Imwitor 948; Peceol < Capmul MCM; Capryol 90. Based on this, Capryol 90, which is the most toxic when compared to the other components, might account for the majority of the cytotoxicity of the NE.

Changing Capryol 90 for other propylene glycol monocaprylates or glycerol monocaprilocaprate did not change the outcome. However, the NE with glyceryl monocaprylate Type II was slightly less toxic (IC_50_ = 0.48%) (Table 3). Nonetheless, Figure 2 demonstrates that this NE lost its small and homogenous droplet size when replacing Capryol 90 with this lipid. Given that the most similar lipids could not replace Capryol 90, other lipids were not tested.

**Figure 3:**
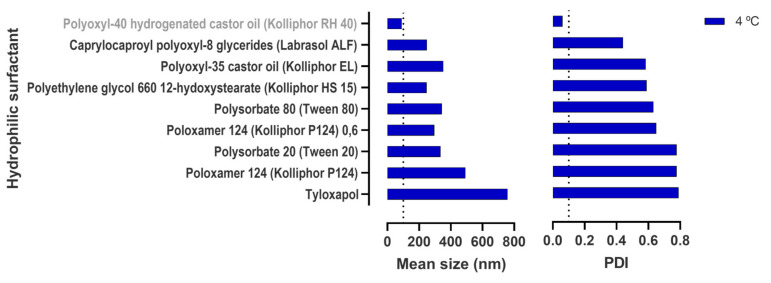
Droplet size characterization of emulsions obtained by changing the hydrophilic surfactant in the selected pre-concentrate. Mean droplet size and polydispersity index (PDI) were measured upon dilution of the refrigerated (4 °C) emulsions. Data correspond to the mean of independent dilutions of a same nanoemulsion.

We also tested many other hydrophilic surfactants for the replacement of Kolliphor RH 40. Still, at least at the proportion being used, neither of them gave comparable results (PDI were over 0.4 and sizes over 200 nm, Figure 3).

**Figure 4:**
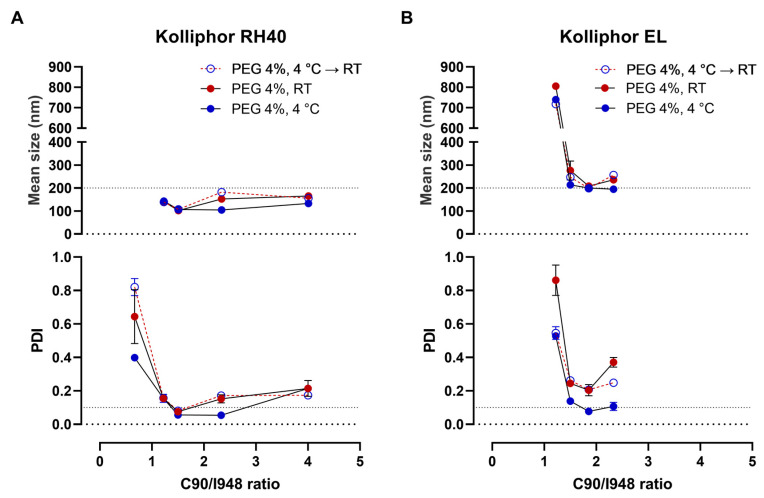
Droplet size characterization of emulsions after varying Capryol 90/Imwitor 948 (C90/I948) ratio. (**A**) Mean droplet size and PDI of nanoemulsions with a fixed amount of Kolliphor RH 40; (**B**) Mean droplet size and PDI of nanoemulsions with a fixed amount of Kolliphor EL. Measurements were made after the emulsions were kept at room temperature (RT), under refrigeration (4 °C), or at room temperature after being kept under refrigeration (4 °C → RT). PEG 4%, polyethylene glycol 4000 aqueous solution at 4% as external phase Data correspond to at least two independent dilutions of the same nanoemulsion.

The proportion of excipients in the formula was also varied. In these experiments, PEG 4000 at 4% was used as the aqueous phase, since it gave the NE a small homogenous droplet size already at room temperature. We started by changing the ratio between Capryol 90:Imwitor 948, originally set at 1.5:1. By doing that, a very small reduction or increase in Capryol 90 in the proportion of the two lipids produced a small but clear increase in both size and PDI, (Figure 4A). However, using refrigeration, the emulsions tolerated better the increase in Capryol 90 in the proportion of lipids, since PDI was still <0.1 with a Capryol 90/Imwitor 948 ratio of 2.333.

Still insisting on the optimization of the compositions containing Kolliphor EL, the variation of the lipids’ ratio was also tested. Slightly better results were obtained after a small increase in Capryol 90 proportion. However, droplet sizes remained higher (about 200 nm), and the emulsion remained dependent on refrigeration to achieve PDI under 0.1 (Figure 4B), even in the presence of PEG in the aqueous phase (Figure 4B).

**Figure 5:**
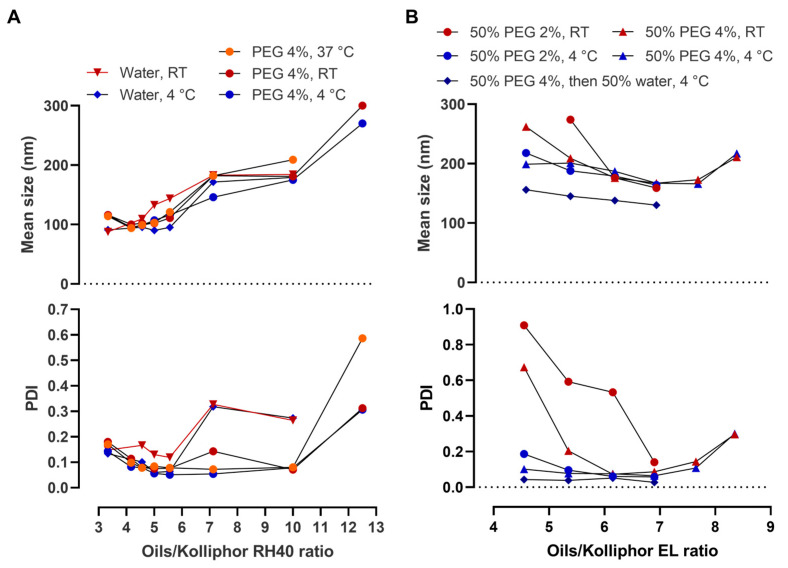
Mean droplet size characterization of emulsions having a varying oils/Kolliphor ratio. (**A**) Data with Kolliphor RH 40; (**B**) Data with Kolliphor EL. Measurements were made after the emulsions were kept at room temperature (RT), under refrigeration (4 °C), or at 37 °C. Data correspond to at least two independent dilutions of the same nanoemulsion.

The effect of varying the proportion between the oils and the hydrophilic surfactant (originally at a proportion of 5:1) was also tested. In formulations containing Kolliphor RH 40, the increase in the ratio of oils/surfactant from five to seven or higher led to the increase in droplets’ mean size. However, in the presence of PEG, PDI values remained low until the ratio was 10 (Figure 5A). On the other sense, the ratio should not be less than four for optimal PDI values.

When using Kolliphor EL as surfactant, the behavior was somewhat different. While the optimum ratio range of oils/surfactant was similar (4.5 to ~7), and refrigeration and PEG were both required, droplet sizes tended to decrease with the increase in oils’ proportion (Figure 5B). Also, the lower mean droplet size and PDI were obtained when preparing the emulsion using a 4% PEG solution and then diluting it again two-fold with water (Figure 5B).

### 3.3. The Nanoemulsion Allows the Incorporation of Both Hydrophilic and Lipophilic Molecules at High Strength

**Figure 6:**
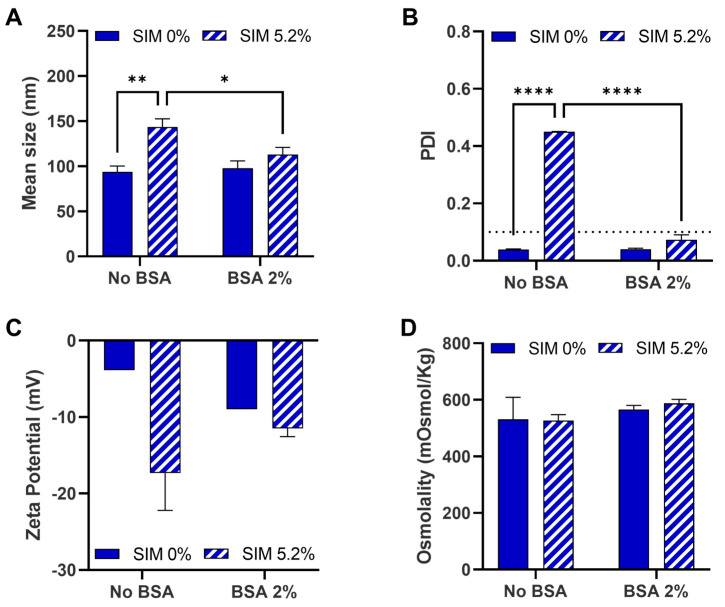
Simvastatin (SIM) and bovine serum albumin (BSA) effect in nanoemulsions’ attributes. Droplet’s mean size (**A**), polydispersity index (PDI, **B**), zeta potential (**C**), and preparation osmolality (**D**) are shown. Measurements were made upon dilution of the refrigerated (4 °C) emulsions. Data correspond to mean ± standard deviation of three independent preparation baches, each measured in two independent dilutions (**A**–**C**) or at least in three replicate measurements (**D**). Statistical significance of differences was tested by two-way ANOVA with Tukey’s multiple comparisons test and given by asterisks: * *p* < 0.05; ** *p* < 0.01; **** *p* < 0.0001.

It was possible to solubilize simvastatin, a lipophilic drug (XLogP3 of 4.7 according to Pubchem), at about 5.2% (*w*/*w*) of the final NE (with 50% aqueous phase). An acidic malate buffer (pH 5) in the aqueous phase was used to maximize the chemical stability of simvastatin [16]. Despite refrigeration of the NE, simvastatin increased the mean droplet size and PDI of the NE, which was significantly reduced back by addition of albumin to the aqueous phase (Figure 6).

Other substances were also solubilized in the NE vehicle at relatively high final strengths (Table 4), mainly very lipophilic ones (cholesterol). Hydrophilic molecules like the prodrug fosphenytoyin sodium can also be added to the aqueous phase at relatively high drug strengths (here the intention was to keep the preparation in a safe osmolality range, but a higher concentration would be possible to obtain).

Next, we also tested if it was possible to remove refrigeration or use other polymers (PEG 4000 and PVP) in simvastatin nanoemulsions while also testing the presence of NaCl in the aqueous phase. While at 4 °C, all tested compositions led to extremely low PDI (≤0.053), at room temperature size and PDI tended to increase and were lower when PEG 4000 was used in the aqueous phase (Table 5).

**Figure 7:**
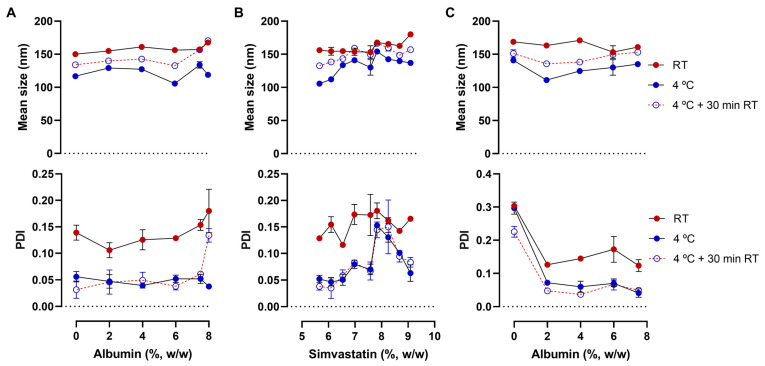
Influence of bovine serum albumin (BSA) and simvastatin (SIM) concentration on nanoemulsions’ mean droplet size. Mean diameter and polydispersity index (PDI) of formulations containing simvastatin at 5.66% (**A**), albumin in the aqueous phase at 6% (**B**) or simvastatin at 7.41% (**C**). Measurements were performed at room temperature (RT), after refrigeration (4 °C), and 30 min at RT after being refrigerated. Data correspond to the mean ± standard deviation of two independent dilutions of one formulation.

The effects of temperature, simvastatin concentration, and albumin concentration were evaluated in more detail. We hypothesized that increasing BSA in the aqueous phase would improve simvastatin solubilization. BSA is able to bind poorly soluble drugs, such as simvastatin, and enhance their solubility. BSA seems to bind to simvastatin in a 1:1 mol ratio due to very strong hydrophobic and hydrogen bond interactions [17]. First, the impact of different BSA concentrations (2–8%) in the water phase on the physical attributes of NE was evaluated (Figure 7A). At room temperature, the NE mean droplet size was very similar among all formulations. On the other hand, larger differences in the PDI values were observed. BSA at 2% originated a lower PDI compared to the NE without BSA. However, PDI remained over 0.1, likely because of pH 5, close to BSAs isoelectric point. At higher BSA concentrations, PDI values increased but were still within the homogenous range (PDI < 0.2). After refrigeration, mean droplet size and PDI decreased in all the formulations, becoming extremely homogenous (PDI < 0.1). The effect of refrigeration on PDI was still visible even if the NE remained for 30 min at room temperature before dilution, although it was sufficient to observe a slight increase in the mean droplet size.

A series of simvastatin increments were then performed to determine the maximum amount of simvastatin capable of being loaded without precipitation when using a BSA 6% solution as the aqueous phase (Figure 7B). Simvastatin increments produced an overall increase in the mean droplet size. At room temperature, all the simvastatin concentrations resulted in PDI values higher than 0.1 but can still be considered homogenous (PDI < 0.2). Once again, refrigeration of the NE produced a decrease in both mean droplet size and PDI, an effect extended for at least 30 min when NE were placed at room temperature again regarding PDI. A final concentration of 9.09% of simvastatin was reached. At higher concentrations of 9.50% simvastatin started to precipitate after the dilution needed for mean droplet size and PDI measurements. Also, at 4 °C, formulations with concentrations higher than 7.41% precipitated in 1–2 days.

In order to further improve the characteristics of NE, BSA was once again tested at different concentrations but, this time, only with 7.41% of simvastatin (Figure 7C). This time it was no longer possible to have a homogenous emulsion without using BSA, even after refrigeration. Even at the lowest concentration (2%) of BSA in the aqueous phase, PDI values decreased from 0.3 to under 0.1 at 4 °C, demonstrating the importance of BSAs presence.

We further evaluated the role of BSA on the physical stability of the refrigerated NE containing simvastatin at 5.66% or 7.41%. Formulations were observed daily until precipitation occurred. To prove the role of temperature in the loss of physical stability, this test was conducted at room temperature and 4 °C (Table 6). With 5.66% simvastatin concentration, both formulations had physical stability (absence of drug precipitation) for at least for 30 days at room temperature and 4 °C. It means that at lower simvastatin concentrations, BSA does not play a major role in increasing physical stability. At 7.41%, the role of temperature in speeding up simvastatin’s precipitation is visible because all formulations achieved better stability at room temperature than at 4 °C. Using BSA at a concentration of 2% did not seem to affect its physical stability because it had exactly the same precipitation day as the NE without BSA (nine days at room temperature and one day at 4 °C). However, with BSA concentrations of 4% and 7.5%, physical stability at room temperature increased to 24 days and over 30 days when at room temperature and 2 days and 6 days at 4 °C, respectively. Despite six days being a short amount of time, the experiment was carried out in microtubes where some solvent evaporation is possible, and the use of BSA at 7.5% could delay precipitation for five more days than when no BSA was used, proving the role of BSA in simvastatin solubilization/stabilization.

### 3.4. Sustained In Vitro Drug Release and In Vivo Performance after Intranasal or Subcutaneous Administration

**Figure 8:**
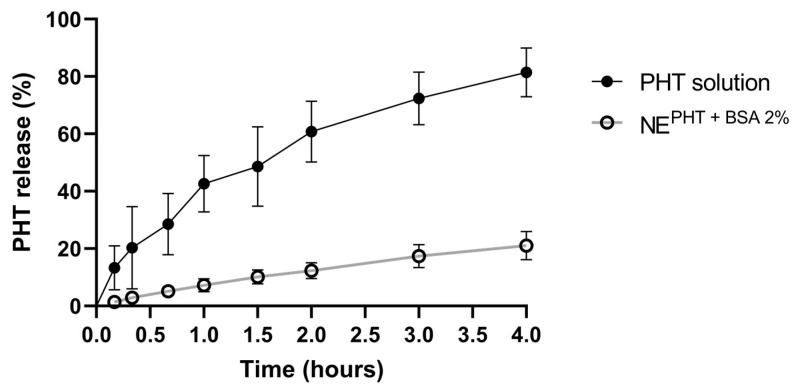
In vitro drug release profile of the developed optimized nanoemulsion, containing bovine serum albumin (BSA) at 2% in the aqueous phase, and phenytoin (PHT) at 3.5 mg/g (NE^PHT + BSA 2%^) in comparison to a solution (in water with 30% Transcutol). Preconcentrate to external phase proportion of 1:1. The preconcentrate was made of Capryol 90: Imwitor 948: Kolliphor RH 40 in a 3:2:1 ratio (weight), and the external phase was 20 mM pH 7 phosphate buffer with BSA.

In vitro drug release from the selected nanoemulsion containing phenytoin as a model drug and albumin at 2% in the aqueous phase was studied (NE^PHT + BSA 2%^, Figure 8). The formulation showed sustained phenytoin release, when compared to a drug solution (0.2 mg/g, in 30% Transcutol), releasing only 30% of phenytoin after 4 h (as opposed to the drug solution, which released over 80%). A sustained drug release capacity could be a means to provide a prolonged therapeutic effect.

**Figure 9:**
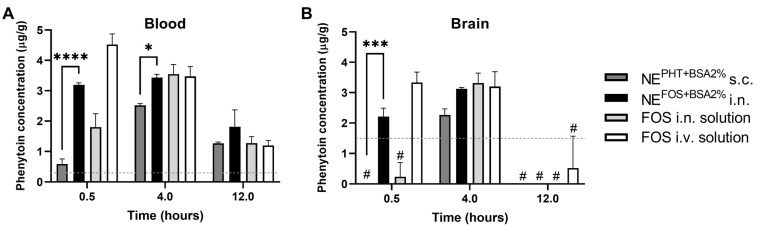
Comparison of phenytoin concentrations in blood and brain after administration of nanoemulsions and solutions of phenytoin and fosphenytoin to mice through different routes. (**A**) Blood phenytoin concentration. (**B**) Brain phenytoin concentration. Administered formulations were either nanoemulsion with bovine serum albumin (BSA) at 2% and phenytoin (PHT) (NE^PHT+BSA2%^), subcutaneously (s.c.) or a nanoemulsion with BSA at 2% and fosphenytoin (NE^FOS+BSA2%^) intranasally (i.n.). Data with a fosphenytoin (FOS) solution [intranasally and intravenously (i.v.)] from previously published results is shown for comparison purposes [15]. The data correspond to mean ± standard deviation. The statistical significance of differences between means was tested by two-way ANOVA with Tukey’s multiple comparisons test and indicated by asterisks only in the comparison of the 2 novel experimental groups: * *p* < 0.05; *** *p* < 0.001; **** *p* < 0.0001. The dashed line represents the limit of quantification (LOQ). # In all samples, phenytoin was detected, but below the LOQ it was considered as equal to zero.

Additionally, in order to demonstrate the relevance of using the NE for hydrophilic drugs and not only lipophilic ones, the fosfenytoin NE was administered intranasally to a small number of mice at comparable conditions to previously published work [15]. The brains and plasma were collected at three time points, and phenytoin (the active form) was quantified and plotted together with previously obtained data for comparison purposes. As demonstrated in Figure 9, phenytoin attained a higher concentration at 30 min after the intranasal administration of the NE than after the intranasal administration of a solution of the same drug. Since the drug strength was not high enough for intranasal administration, a NE containing phenytoin was also subcutaneously administered at a similar dose, demonstrating, however, that subcutaneous administration is less favorable (lower concentrations were achieved at 30 min and 4 h) than intranasal administration.

## 4. Discussion

Several related low energy methods of NE production have been described [18]. The most closely related to the process we used here with this novel NE is the reverse phase emulsification method, also named phase inversion emulsification [19]. We claim this because we observed smaller and more homogenous droplet size by adding the aqueous phase in two steps (a very low amount first to force the formation of a water-in-oil emulsion, and then the rest to inverted it to oil-in-water) than in a single step. Since long ago, this strategy has been used in the traditional continental method of oral emulsion preparation to obtain finer droplet dispersions [20]. This is also one of the reasons why we describe our system as a NE and not a microemulsion, since for microemulsions formation the order of excipients addition is not supposed have an effect.

Temperature is known to induce phase inversion of some emulsions with neutral surfactants (phase inversion temperature or PIT method [21]), and refrigeration is known to increase emulsions’ physical stability. However, a particular composition that spontaneously forms smaller and more homogeneous droplets upon refrigeration without phase inversion has, as far as we know, not been previously described. The fact that after dilution (at least 10-fold) the small and homogeneous size is maintained at room temperature is likely due to the slower rearrangement of droplets at a greater distance.

It was also very surprising that the optimum mass proportion of oils to hydrophilic surfactant was between four and six to one. In fact, it is important to emphasize that the optimized preconcentrate has a composition of 83% of lipids or, more precisely, water-insoluble surfactants, and only 17% of a hydrophilic surfactant, which has no correspondence in the classification of lipid based formulations by Colin Pouton [22]. The explanation of why the aqueous dispersion of this particular composition has this behavior and how refrigeration or the composition of the aqueous phase can modulate a droplet’s size and PDI is beyond the scope of our work. What has been shown, however, is how narrow the optimum design space seems to be.

Regarding the potential utility of the NE or of its preconcentrate, much remains to be explored in future works. One of the drugs that was demonstrated to solubilize at high strength in this vehicle was simvastatin, a lipophilic statin with low oral availability. This is a drug with pleiotropic effects and many potential applications beyond the control of dyslipidemia, like anti-cancer [23], anti-fibrotic [24], neuroprotective [25,26], and bone regeneration [27] applications, among others. Some of these applications could benefit from an alternative liquid formulation for, for example, intranasal administration, which has the potential to enhance brain delivery [28]. However, a limiting factor should be the relative cytotoxicity of the NE lipids Capryol 90 and Imwitor 948 compared to other lipids, as shown here. In fact, both Capryol 90 and Imwitor 948 are approved for cutaneous and oral administration and not for parental use. Therefore, the cutaneous and oral use of this NE, or of the preconcentrate as a self-nanoemulsifying drug delivery system, should not pose safety concerns since all excipients are well established and approved for these routes. Therefore, many possible applications could be envisioned in cosmetic or oral medicinal products. However, their safety for intranasal, ophthalmic, vaginal or parenteral delivery must still be established. For these routes, it is likely that there are limitations to the concentration/dose of the vehicle itself that can be safely used. Nevertheless, given the high content in Capryol 90:Imwitor 948, the studied NE might have the potential to provide a means to obtain a high concentration of lipophilic substances with a good solubility profile in this oil mixture. When compared to other oils, Capryol 90, in higher proportion in our formula, has shown to be the better solubilizer (among those tested) for very lipophilic drugs such as simvastatin (105 mg/mL [29]), clofazimine (18 mg/mL, [30]), luliconazole [~75 mg/mL [31]), terconazole (116 mg/mL [32]), tolvaptan (11 mg/g [33]), and even novel drug candidates such as JIN-001 (41 mg/mL [34]) and AC1497 (45 to 50 mg/mL [35]), to mention just a few examples. Precipitation upon dilution is not expected to occur since no water-miscible co-solvents account for the initial drug solubilization, and droplet size does not reduce upon dilution and is shown to provide a slow release of phenytoin used as the model drug. Furthermore, the small size and optimal homogeneity are in favor of long-term physical stability for these compositions. As we have demonstrated, it can also promote the mucosal absorption of hydrophilic substances. This effect was similar to what was previously observed by us with a microemulsion of fosphenytoin [36].

## 5. Conclusions

A novel composition with a high lipid/surfactant ratio was described for the first time, comprising Capryol 90 and preferentially Imwitor 948 and Kolliphor RH 40 in the optimum proportion of 3:2:1. This composition (preconcentrate) surprisingly formed an extremely homogeneous NE (PDI < 0.1) upon addition of the aqueous phase at about 50% using only mild manual agitation, not allowing much variation of excipients and their proportions for optimal physical attributes. This novel NE could be most suitable for the dissolution of lipophilic drugs such as simvastatin or for increasing the absorption of hydrophilic ones like fosphenytoin. Its low PDI, along with small droplet size, is expected to contribute favorably to the formulation’s physical stability during storage. Using plain water, refrigeration was necessary to obtain the lowest PDI values, but several hydrophilic polymers could be used in the aqueous phase to promote the greatest homogeneity at room temperature or even higher temperatures. Future work will explore the usefulness of this novel formulation in the delivery of simvastatin and other drugs.

## 6. Patents

WO2022259205A1—WIPO (PCT). Self-emulsifying composition, production methods and uses thereof. Inventors: Adriana Oliveira dos Santos, Mariana Carvalho Fernandes, Patrícia Sofia Cabral Pires, Gilberto Lourenço Alves, Ana Carolina Maricoto Fazendeiro, Francisca Matos Silva Pereira Nina, Maria De Fátima Da Silva Ferreira Gomes, Lina Isabel Esteves Rodrigues. Publication date: 15 December 2022. International Application Number: PCT/IB2022/055385. International filling date 9 June 2022; Applicant: University of Beira Interior. Status: pending.

## Figures and Tables

**Table 1:** Composition and half-inhibitory concentration (IC_50_) of different nanoemulsions obtained by varying the secondary oil (16.7% *w*/*w*). All the NE also contained Carpyol 90 (25%) and Kolliphor RH 40 (8.3%). IC_50_ is expressed as % (*w*/*v*) of preconcentrate.

Secondary Oil		Aqueous Phase		IC50 (%)
Compendial Name	Brand Name	BSA 4% * (%)	PEG 4% (%)	
Glycerol mono-oleate	Imwitor 948	50		0.037
Glycerol mono-oleate	Peceol	50	-	0.027
Isopropyl myristate	Kollicream IPM	50	-	0.051
Glycerol mono-oleate	Imwitor 948	-	50	0.041
Glycerol monocaprilocaprate	Capmul MCM	-	50	0.021
Soybean oil	-	-	50	0.060
Glycerol monolinoleate	Maisine CC	-	50	0.026
Vitamin E acetate	-	-	50	0.037

* Malate buffer pH 5; BSA, bovine serum albumin; PEG, poly ethylene glycol 4000.

**Table 2:** Lipidic composition and half-inhibitory concentration (IC_50_) of different microemulsions prepared with different oils. Formulations are ranked from the least toxic to the most toxic. Other excipient’s concentration (Kolliphor RH 40:Transcutol HP: and Water) was 40%:40%:10% (*w*/*w*). IC_50_ is expressed as % (*w*/*v*) of the preconcentrate without accounting for water.

Lipid (10%)		IC_50_
Compendial Name	Brand Name	
No lipid (extra 10% water)		32.6
Vitamin E acetate		12.0
Medium chain triglycerides	Miglyol 812	10.6
Bis-diglyceryl polyacyladipate-2	Softisan 64S	10.5
Soybean oil		10.2
Propylene glycol dicaprylocaprate	Labrafac PG	7.34
Isopropyl myristate	Kollicream IPM	7.04
Glycerol monolinoleate	Maisine CC	6.71
Glycerol mono-oleate	Imwitor 948	1.67
Glycerol mono-oleate	Peceol	0.52
Glycerol monocaprilocaprate	Capmul MCM	<0.16 (0.08)
Propylene glycol monocaprylate Type II	Capryol 90	<0.16 (0.08)

**Table 3:** Composition and half-inhibitory concentration (IC_50_) of different NE obtained by varying the main oil (25%). The rest of the composition was Imwitor 948 (16.7%), Kolliphor RH 40 (8.3%) and as aqueous phase a solution of PEG 4000 at 4% (50%). IC_50_ is expressed as % (*w*/*v*) of the preconcentrate without accounting for the aqueous phase.

Main Oil		IC50 (%)
Compendial Name	Brand Name	
Propylene glycol monocaprylate NF (Type II)	Capryol 90	0.041
Propylene glycol monocaprylate NF (Type II)	Capmul PG-8-70 NF	<0.16
Propylene glycol monocaprylate	Capmul PG-8	<0.16
Propylene glycol monocaprylate Type I	Capryol PGMC	<0.16
Glyceryl monocaprylate Type II	Capmul 808G EP/NF	0.48
Glycerol monocaprilocaprate	Capmul MCM	0.08

**Table 4:** Composition and mean droplet size characterization of nanoemulsions solubilizing chemicals with different lipophilia. Preconcentrate to external phase proportion of 1:1, with the preconcentrate composition in excipients being Capryol 90: Imwitor 948: Kolliphor RH 40 in a 3:2:1 ratio (weight), and the external phase a BSA aqueous dispersion (at 2 or 4% in 20 mM phosphate buffer, pH 7). In the case of fosphenytoin sodium solubilization, it was dissolved in the aqueous phase and the pH titrated to neutrality.

External Phase’s BSA Concentration	Solubilized Molecule	XLogP3 *	%	RT		4 °C	
				Mean Size (nm)	PDI	Mean Size (nm)	PDI
2%	Cholesterol	8.7	5	110	0.064	n.d.	n.d.
2%	Cholesterol	8.7	7.5	138	0.097	111	0.075
4%	Segesterone Acetate	2.9	1.12	100	0.074	80	0.092
2%	Phenytoin	2.5	0.35	94	0.042	90	0.029
4%	Phenytoin	2.5	0.35	124	0.144	89	0.021
4%	Fosphenytoin sodium	0.6 **	4.25	85	0.177	61	0.082

BSA—bovine serum albumin; CHOL—cholesterol; FOS—fosphenytoin; n.d.—not determined; PDI—polydispersity index; PHT—phenytoin; RT—room temperature; * LogP valued are according to Pubcehm; ** this is not the maximum concentration, just the one required for an isotonic preparation.

**Table 5:** Composition and mean droplet size characterization of additional simvastatin emulsions having a preconcentrate to external phase proportion of 1:1. The preconcentrate was made of Capryol 90: Imwitor 948: Kolliphor RH 40 in a 3:2:1 ratio (weight), and the external phase was made of saline malate buffer (pH 5, containing 0.4% malic acid and 0.8% sodium chloride) with either bovine serum albumin (BSA), poly ethylene glycol 4000 (PEG) or polyvinylpyrrolidone (PVP) at 4%.

Simvastatin (%)	Polymer in the aq. Phase	RT		4 °C	
		Mean Size (nm)	PDI	Mean Size (nm)	PDI
5.20	BSA 4%	160.4	0.104	102.8	0.041
4.56	PEG 4%	106.2	0.060	101.4	0.042
4.46	PVP 4%	110.1	0.192	101.8	0.053
5.66	PVP 4%	-	-	115.1	0.046

BSA—bovine serum albumin; PEG—poly ethylene glycol 4000; PDI—polydispersity index; PVP—polyvinylpyrrolidone; RT—room temperature.

**Table 6:** Time at which precipitation was observed at room temperature and 4 °C in different nanoemulsions containing albumin (BSA) at different concentrations (0%, 2%, 4%, and 7.5%) and simvastatin at 5.66% or 7.41%. Preconcentrate to external phase proportion of 1:1. The preconcentrate was made of Capryol 90: Imwitor 948: Kolliphor RH 40 in a 3:2:1 ratio (weight), and the external phase was made of malate buffer (pH 5) with BSA.

Nanoemulsions		Time for Precipitation (Days)	
Simvastatin	BSA	Room Temperature	4 °C
5.66%	0%	>30	>30
	2%	-	>30
	4%	-	>30
	7.5%	>30	>30
7.41%	0%	9	1
	2%	9	1
	4%	24	2
	6%	-	3
	7.5%	>30	6

## Data Availability

Data is contained within the article or Supplementary Material in the summarized form (graphs and tables). The corresponding raw data are available on request from the corresponding author.

## Supplementary Materials

The following supporting information can be downloaded at: https://www.mdpi.com/article/10.3390/pharmaceutics15071878/s1, Figure S1: Droplet size characterization of the initial screen emulsion formulas before and after extrusion.
