# Supplementary material for: Innovative Aqueous Nanoemulsion Prepared by Phase Inversion Emulsification with Exceptional Homogeneity
Source: Pharmaceutics. 2023 Jul 4;15(7):1878. doi: 10.3390/pharmaceutics15071878 (PMC10384498; doi:10.3390/pharmaceutics15071878)
Supplement: Supplementary file 1 [file pharmaceutics-15-01878-s001.zip › pharmaceutics-2449662-supplementary.pdf]

pharmaceutics

MDPI

Supplementary data

Innovative aqueous nanoemulsion prepared by phase inversion emulsification with exceptional homogeneity.

Patrícia C. Pires [1,2,3,†], Mariana Fernandes [1,4,†], Francisca Nina [1,4,†], Francisco Gama [1,4,†], Maria F. Gomes [1,4], Lina E. Rodrigues [1,4], Sara Meirinho [1,4], Samuel Silvestre [1,5], Gilberto Alves [1,4], Adriana O. Santos [1,4*]

[1] CICS-UBI—Health Sciences Research Centre, University of Beira Interior, Av. Infante D. Henrique, 6200-506 Covilhã, Portugal;
[2] Department of Pharmaceutical Technology, Faculty of Pharmacy, University of Coimbra, 3000-548 Coimbra, Portugal
[3] REQUIMTE/LAQV—Associated Laboratory for Green Chemistry of the Network of Chemistry and Technology, Faculty of Pharmacy, University of Coimbra, 3000-548 Coimbra, Portugal
[4] Faculty of Health Sciences, University of Beira Interior, Av. Infante D. Henrique, 6200-506 Covilhã, Portugal
[5] Faculty of Sciences, University of Beira Interior, Rua Marquês d'Ávila e Bolama, 6201-001 Covilhã, Portugal
* Correspondence: asantos@fcsaude.ubi.pt
† These authors contributed equally to this work.

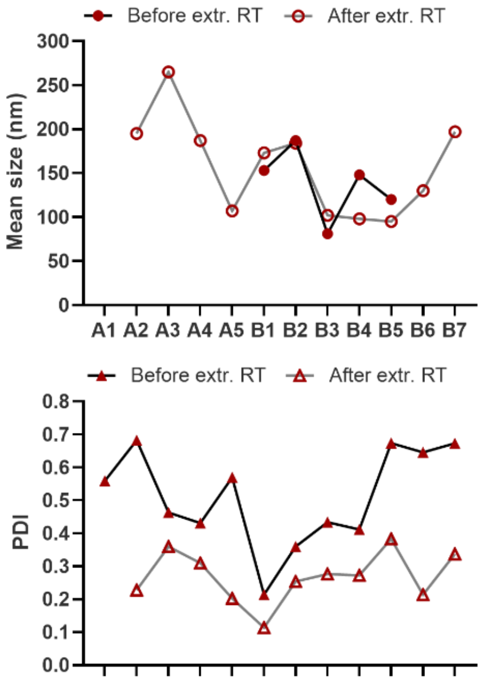

	Formula code	A1	A2	A3	A4	A5	B1	B2	B3	B4	B5	B6	B7
Composition (%)	Capryol 90	0	0	0	0	0	15	20	18	20	23	25	27
	Imwitor 948	20	18	17	15	10	10	5	0	0	0	0	0
	Kolliphor RH40	5	10	10	10	10	5	5	10	8	8	5	3
	Transcutol	0	3	3	3	5	0	0	3	3	0	0	0
	Water	75	70	70	73	75	70	70	70	70	70	70	70

Figure S1. Droplet size characterization of the initial screen emulsion formulas before and after extrusion. Extrusion (Avanti Polar Lipids Mini Extruder®) through polycarbonate membranes with a pore of 200 nm (19 mm, Whatman® Nuclepore™ Track-Etched, Sigma-Aldrich, Steinheim, Germany) was used a means of homogenization known as premix membrane emulsification. Some mean size values before extrusion are missing due to insufficient quality in cumulants analysis because of high heterogeneity in size. PDI—polydispersity index; RT—room temperature.
